# Representation of amplified speech at cortical level in good and poor hearing aid performers^☆^

**DOI:** 10.1016/j.bjorl.2019.02.010

**Published:** 2019-04-23

**Authors:** Hemanth Narayan Shetty, Manjula Puttabasappa

**Affiliations:** All India Institute of Speech and Hearing, Department of Audiology, Karnataka, India

**Keywords:** Annoyance, Amplification, Aging, Aborrecimento, Amplificação, Envelhecimento

## Abstract

**Introduction:**

Hearing aid users reject their own hearing aid because of annoyance with background noise. The reason for dissatisfaction is located anywhere from the hearing aid microphone to the integrity of neurons along the auditory pathway. In this preview, the output of hearing aid was recorded at the level of ear canal and at auditory cortex in good and poor hearing aid users, who were classified using acceptable noise level.

**Objective:**

To study the representation of amplified speech in good and poor hearing aid performers.

**Methods:**

A total of 60 participants (age ranged 15–65 years) with moderate bilateral sensorineural hearing impairment grouped into good (*n* = 35) and poor (*n* = 25) hearing aid performers. Gap detection test and aided SNR 50 were administered. In addition, ear canal acoustic measures and cortical auditory evoked potentials were recorded in unaided and aided conditions at 65 dB SPL.

**Results:**

Hearing aid minimally alters temporal contrast of speech reflected in envelope difference index. Although having similar temporal impairment, acoustic characteristics of amplified speech sounds and SNR 50 scores from both groups, the aided cortical auditory evoked potentials surprisingly showed significant earlier latencies and higher amplitudes in good performers than poor performers. In addition, good and poor performers classified based on annoyance level was predicted by latencies of 2N1 and 2P2 components of acoustic change complex. Further, a follow-up revealed hearing aid use has relation with acceptance towards noise.

**Conclusion:**

Participants who are willing to accept noise from those who are not willing to accept noise have subtle physiological changes evident at the auditory cortex, which supports the hearing aid usage.

## Resumo

**Introdução:**

Usuários de aparelhos auditivos rejeitam seu próprio aparelho pelo incômodo relacionado ao ruído de fundo. O motivo da insatisfação varia desde o microfone do aparelho auditivo até a integridade dos neurônios ao longo da via auditiva. Nesta análise prévia, a saída da prótese auditiva foi registrada no nível do meato auditivo externo e no córtex auditivo em bons e maus usuários de prótese auditiva, que foram classificados com um nível de ruído aceitável.

**Objetivo:**

Estudar a representação da fala amplificada em bons e maus usuários de aparelhos auditivos.

**Método:**

Foram examinados 60 participantes (de 15 a 65 anos) com deficiência auditiva neurossensorial bilateral moderada divididos em bons (n = 35) e maus (n = 25) usuários de aparelhos auditivos. Teste de detecção de *gap* e SNR 50 assistido foi administrado. Além disso, as medidas acústicas do meato externo e os potenciais evocados auditivos corticais foram registrados em condições não assistida e assistida a 65 dB NPS.

**Resultados:**

O aparelho auditivo altera minimamente o contraste temporal da fala, refletido no *envelope difference index*. Embora tenham uma deficiência temporal similar, as características acústicas de sons de fala amplificados e escores do SNR 50 de ambos os grupos, os potenciais evocados auditivos corticais assistidos surpreendentemente demonstraram latências iniciais significativas e maiores amplitudes em bons do que em maus usuários. Além disso, os bons em comparação com os maus usuários, classificados com base no nível de incômodo, foram previstos pelas latências dos componentes 2N1 e 2P2 do complexo de alteração acústica. Além disso, os resultados do seguimento revelaram que o uso de aparelhos auditivos têm relação com a aceitação do ruído.

**Conclusão:**

Os participantes dispostos a aceitar o ruído, quando comparados com aqueles que não estão dispostos a aceitá-lo, apresentam evidência de sutis alterações fisiológicas no córtex auditivo, que apoiam o uso da prótese auditiva.

## Introduction

Older adults with hearing impairment find it difficult to process vowel-formant discrimination due to a deficit in temporal encoding.^1^ One of the rehabilitative devices available to them is the hearing aid. Kochkin^2^ reported on an average 62.3% are dissatisfied with hearing aids of which 25.3% reject their hearing aid because of background noise. Acceptable noise level is a subjective measure to test the annoyance by assessing the patient's willingness to accept background noise; its results are related to hearing aid use.^3^ Nabelek et al.^4^ demonstrated that the acceptable noise level (ANL) predict successful and unsuccessful hearing aid users with 85% accuracy. Further, variability in the successful/unsuccessful use of a rehabilitative device might probably be due to the processing parameters of the hearing aid, and/or at the interaction between the output of the hearing aid and its acoustic parameters relayed up to the cortical level.^5^, ^6^ Though individuals have a similar type, degree, and configuration of hearing loss the reason for satisfaction/rejection with a similar advanced hearing device is still unclear. Thus, it would be interesting to note how the speech syllables are processed at the ear canal and at auditory cortex in a hearing aid user. Speech signals recorded at ear canal^7^ to which temporal contrast between unaided and aided version of stimulus can be precisely quantified by an objective method envelope difference index (EDI).^8^, ^9^ The rationale here is that some changes in speech perception can be explained by how the listeners process acoustic changes in the temporal structure of the sound caused by the hearing aid and that is measured via the EDI. In addition, signal level and noise level and its ratio are equally important.^10^ This is because during amplification to a desired signal there is a likelihood chance of amplifying the ambient noise too.^11^ This means crucial cues are less available for hearing impaired individuals. However, accessing those available cues indeed require high SNRs for them to understand speech. Thus, utilizing speech signal as input in PTM measurement facilitates in knowing the way in which a hearing aid represents the inherent features of speech in the ear canal of the participant. In a few individuals, the inherent cues are unavailable but they still manage to comprehend the information through redundancy cues. Whereas, in some individuals a critical cue appeared to be available at the output of the hearing aid the errors exhibited by them cannot be resolved by acoustic analysis of hearing aid output alone. Thus, it is for this reason that the study concerning the representation of acoustic cues at the cortical level becomes imperative.^12^ Cortical auditory evoked potentials (CAEPs) assess the neural detection of sound at the auditory thalamic-cortical level^13^ CAEPs are elicited by clicks,^14^ tones^15^ and synthetic^16^ and natural consonant vowel stimuli.^17^, ^18^

Thus, it is hypothesized that though study participants have similar degree of hearing loss, temporal ability, minimally altered temporal envelope after processing through the hearing aid, an annoyance level toward noise in them may show difference in representation of amplified speech at cortical level in good and poor hearing aid performers. The following objectives were formulated: (1) to evaluate gap detection threshold, temporal alteration induced by hearing aid using EDI, SNR 50 and encoding of speech stimuli at cortical level using late latency response and acoustic change complex in good and poor hearing aid performers; (2) to find the relationship between signal to noise ratio of recorded output and latency; and amplitude of CAEPs and (3) to investigate which of these measures predicts good and poor hearing aid performers.

## Methods

A factorial research design was utilized as the assigned independent variable was group (with two levels) made based on annoyance level toward noise. The hearing aid outcome was the dependent variable, which was assessed through behavioral, psychoacoustic and electrophysiological approaches.

### Participants

Sixty patients who had bilateral moderate sensorineural hearing loss with flat configuration were recruited for the study. Flat configuration was operationally defined as the difference between the least and highest air conduction thresholds of the test ear being less than 20 dB from 250 Hz to 8000 Hz.^19^ Flat configuration with moderate hearing loss group, is the one that understand the speech contributed from wide range of different frequencies than any other configuration of hearing loss. The age range of the participants was 15–65 years (mean age = 49.45 years). Speech identification scores (SIS) at 40 dB SL (ref: Speech reception threshold, SRT) were greater than or equal to 75%. Those participants who had normal middle ear status indicated by ‘A’ type tympanogram were included. Those participants having a latency difference of V peak of auditory brainstem responses was less than 0.8 ms between the two repetition rates (11.1 s and 90.1 s presented at 90 dB nHL) were included in the study. They were naïve hearing aid users with no self-reported history of other otological and neurological problems. The participants were grouped into good or poor hearing aid performers using acceptable noise level (ANL). Those participants who obtained an ANL score of ≤7 were considered as good hearing aid performers (*n* = 35) and a score of ≥13 were considered as poor hearing aid performers (*n* = 25). The participants were excluded if their ANL was between 7 and 13.^4^

Informed consent was obtained from all individual participants included in the study. All procedures performed in studies involving human participants were in accordance with the ethical standards of the AIISH institutional research committee (SH/AEC/HN/2013-14).

### Acceptable noise level

The recorded Kannada passage was routed to the loudspeaker through the auxiliary input of the audiometer at the level of speech recognition threshold (SRT). Gradually, the level was adjusted in 5 dB-steps up to the level of most comfortable level (MCL) and then in smaller steps size of +1 and −2 dB, until the MCL of the participant was established reliably. After the MCL was established, a speech noise was introduced at 30 dB HL. The level of the speech noise was increased in 5 dB-steps initially, and then in 2 dB-steps, to a point at which the participant was willing to accept the noise without becoming tired or fatigued while listening to and following the words in the story. The maximum level at which he/she could accept or put up with the noise without becoming tired was considered as the (BNL). ANL is calculated by subtracting BNL (dB HL) from MCL (dB HL) and based on the scores of ANL, each participant of clinical group was classified as good (≤7) and poor (≥13) hearing aid performers.

### Gap detection test

Temporal processing ability from each of the study participant was obtained binaurally from the gap detection test (GDT). Maximum likelihood procedure (MLP) m-code stored in MATLAB (2009B) was used to deliver the gap in three-interval force choice method. The output of a personal laptop was routed through the audiometer. Output of the audiometer was presented through the headphone at participant's most comfortable level. The paradigm comprised of three blocks of broad band noise having a duration of 500 ms each and having inter block intervals of 200 ms. In one among three blocks of noise the gap used to be present at center. Further, the occurrence of gap in one of the block will be randomized in each trial. The step size in gap detection test utilized was 0.5 ms. The duration of gap in succeeding trial will be decided based on participant response in the previous trial. Bracketing method (+0.5 ms and −1 ms) was used to detect the minimum gap will be noted down.

### Recording hearing aid output at ear canal

The Microtech radius ‘2’ behind the ear hearing aids was used. According to manufacturer's specification the frequency range of each of this hearing aid extended from 210 to 5400 Hz. The peak full-on gain was 58 dB and 56 dB and high-frequency average full-on gain was 49 dB was 50 dB, respectively. The equivalent input noise was 10 Db SPL and 9 dB SPL. The signal processing delay of the hearing aids was 4.4 ms and 4 ms. Further, the attack and release times were 10–15 ms and 30–35 ms, respectively for an input of 2000 Hz tone. The functioning of both the hearing aids were ensured at the beginning of the data collection and repeated every 3 months till the completion of data collection.

Each participant was fitted with the digital BTE test hearing aids binaurally using a custom-made soft shell mold. The hearing aid was programmed using NAL-NL1 prescriptive formula with noise reduction circuit being activated. The real ear measurement was carried out using FONIX 8000 hearing aid analyser to optimize the gain of the each hearing aid to match the target gain objectively. In addition, it was ensured that from each ear the aided thresholds from 500 Hz to 4 kHz in octaves were within aided speech spectrum. Each participant was scheduled to visit the clinic after one month of their hearing aid usage. A data logging option in the software was utilized to assess an average number of hours in a month the hearing aid being used by the client was documented.

**Figure 1:**
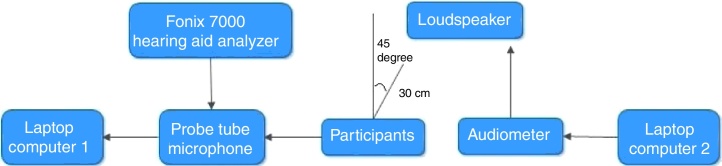
Instrumentation used to record CV syllables in the ear canal of the participant.

The output of the hearing aid for /da/ and /si/ stimuli at the ear canal was measured, in both unaided and aided conditions. The output at the ear canal from the test ear was chosen equally from either right or left ear in random order. The output of the laptop computer 2 was connected to the auxiliary input of audiometer to present the recorded CV syllables /da/and /si/ stimuli at 65 dB SPL. The speech output from the audiometer was delivered through a loudspeaker. The output of these stimuli (/da/ and /si/ stimuli, in both unaided and aided conditions) was subsequently digitally recorded using a laptop computer 1 installed with Praat software (version 5.1.29), using a sampling frequency of 44.1 kHz and 16 bit resolution. Fig. 1 depicts the block diagram to record CV stimuli in the ear canal of the participant. The two CV stimuli recorded from the probe tube microphone, in unaided and aided conditions, were subjected to envelope difference index (EDI). The EDI value ranges from 0 to 1 where 0 indicate no difference in envelope between unaided and aided version of stimuli and 1 indicate temporal alteration. Further, a level of signal, noise level and signal to noise ratio were calculated for /si/ stimulus using matlab. The recorded signal was edited 300 ms analysis windows immediately preceding the /si/ stimulus. The rms was calculated using matlab function in the 300 ms analysis windows immediately preceding the stimulus /si/. It is considered as baseline or ambient noise level rms (A). In addition, rms of the signal /si/ was calculated and it is accumulated with the signal plus an ambient noise level (B). Further, rms of the signal (B) was subtracted from the rms of signal (A) to obtain rms of signal/si/ level (C). The following formula was executed in matlab to calculate the SNR.

It is calculated by the ratio between the signal power and the noise power: SNR = P(C)/P(A). SNR is also usually represented in decibels Sound pressure level (dB SPL): SNR = 20 log (P(C)P(A))(dB).

### Acquiring the acoustic change complex

Each participant fitted with binaural hearing aids was seated comfortably in an armrest reclining chair. The electrode sites were cleaned with skin preparing gel. Disk type silver coated electrodes were placed using conduction gel at the test sites. The non-inverting electrodes (+) were placed on the Cz, Fpz, C3, and C4, the ground electrode was on upper forehead (Fpz) and the inverting electrode (–) was placed on nose. Horizontal (electrodes placed left and right outer canthi of eyes) and vertical (electrodes placed above and below eye) electro ocular responses were eliminated from the recoding. It was ensured that the electrode impedance was less than 5k Ω for each of the electrodes and that the inter-electrode impedance was less than 2k Ω.

The loudspeaker was placed at 0° Azimuth located at the calibrated position of 1 m distance. The height of the loudspeaker was at the level of ear of the participant. The participant was instructed to ignore the stimulus and watch a movie that was muted and played through a battery-operated laptop computer. He/she was also asked to minimize the head movement. The PC-based evoked potential system, Neuroscan 4.4 (Stim 2-version 4.4), controlled the timing of stimulus presentation and delivered an external trigger to the evoked potential recording system, Neuroscan (Scan 2-version 4.4). To allow for a sufficient refractory period within the stimulus sweep, while minimizing the total recording time, an inter-stimulus interval (ISI) of 700 ms from offset to onset of the next stimulus was included for recording LLR to/da/ stimulus. However, for recording unaided and aided acoustic change complex, /si/ stimulus was used with an ISI of 700 ms.

The recording was initiated once a stable EEG was obtained. The ongoing EEG was online band-pass filtered from 0.1 to 100 Hz with 12 dB/octave roll-off and continuously recorded. This was stored to disk for offline analysis. LLR recorded for 250 sweeps each in alternative polarities, delivered in a homogeneous train using stimulus presentation software Neuroscan 4.4 (Stim 2-version 4.4). A similar procedure was repeated to record the unaided and aided Acoustic Change Complex (ACC) for /si/ stimulus. The order of stimuli while testing on each participant was counter-balanced. Each potential (LLR and ACC) was recorded twice for reliability. The continuous EEG data were epoched over a window of 800 ms for each stimulus (including a −100 ms pre-stimulus period and a 700 ms post-stimulus time). The epoched waveforms were corrected for baseline. After eye-blink rejection, the remaining sweeps were averaged and filtered off-line from 1.0 Hz (high-pass filter, 24 dB/octave) to 30 Hz (low-pass filter, 12 dB/octave). All artifacts exceeding ± 75 μV were rejected while averaging the response for alternative polarity. It was found that unaided response was absent as the stimulus intensity of 65 dB SPL failed to elicit the response. Thus, aided response was analyzed from the good and poor hearing aid performers. At the cortical level, the response was recorded from four electrode sites ‘C3’, ‘Cz’, ‘C4’ and ‘Fpz’. The electrode site from which higher amplitude was recorded was utilized to investigate the representation of speech syllables.

### Speech in noise test

Each participant was fitted with the digital BTE test hearing aids binaurally. The speech stimuli used were standardized Kannada sentences developed by Geetha et al.^20^ Ten phonemically equivalent sentences have five target words in each were utilized to add noise at different SNRs. Speech shaped noise having aspectrum similar to that of standardized sentence was prepared. For each sentence, root mean square (RMS) was identified and then noise was added at desired SNR. The ten sentences were mixed with speech shaped noise at different signal to noise ratios ranged from +12 dB to −6 dB SNR in 2 dB step size. The onset of noise was started 500 ms before the onset of each sentence and continued for 500 ms after the offset of the sentence. A smooth ramp of 50 ms (rise and fall time) was made to the noise using cosine function to avoid unintended effects. The following formula was used to add noise to each sentence in AUX viewer software.

SNR=wave(filename)@rms≫500+ramp(wave(noise)@rms,20)

Ten sentences embedded at different SNRs were randomized. Each sentence was presented at 65 dB SPL in aided condition. The participants were instructed to repeat the sentence heard. The SNR level at which the testing started (*L*) and number of correctly recognized target words in each sentence was noted down. The total number of target words from all sentences was added (*T*). Also, the total number of words per decrement (*W*) and SNR decrement step size in each sentence (*d*) were noted down. The obtained values were substituted to the given equation adapted by Spearman–Karber to determine SNR 50%. The equation below was used to calculate the SNR 50 (50 point = *L* + (0.5*d) − *d* (*T*)/*W*).

## Results

**Figure 2:**
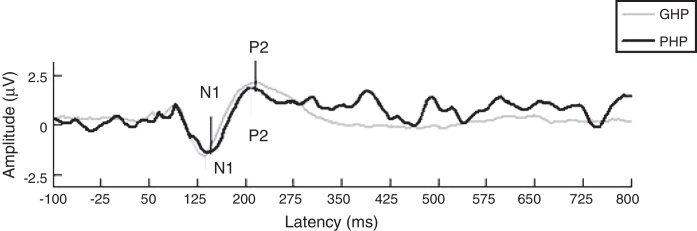
Grand average waveform of LLR obtained from GHP and PHP is represented. The latencies of N1 and P2 were slightly earlier in GHP than PHP.

The mean and Standard Deviation (SD) of gap detection threshold (in ms) was less in good hearing aid performers (12.95 ± 0.43) than poor hearing aid performers (12.18 ± 0.53). To examine the effect of groups on gap detection threshold, an independent samples t test revealed no significant difference between groups on GDT [*t*(58) = 1.07, *p* = 0.305]. This infers temporal processing ability is similar in both groups. In addition, the data of EDI were analyzed for each stimulus, which was recorded from participants’ ear canal of good and poor hearing aid performers. The Mean (M) and Standard Deviation (SD) of EDI for /da/ stimulus was 0.31 ± 0.04 in GHP and 0.30 ± 0.36 in PHP. For /si/ stimulus the value of EDI was 0.36 ± 0.06 in GHP and 0.34 ± 0.26 in PHP. An independent samples *t* test revealed that there was no significant difference on EDI between GHP and PHP for /da/ [*t*(58) = 0.81, *p* = 0.430] and /si/ [*t*(58) = 0.76, *p* = 0.459] stimuli. This infers though a difference in EDI was not observed between groups the output of hearing aid induces a minimal temporal alteration on each stimulus. Further, the aided representation at cortical response for two stimuli /da/ (slope of LLR) and /si/ (ACC) were analyzed. It was noted that though the slope of N1-P2 was steeper in GHP (0.057 ± 0.03) than in PHP (0.047 ± 0.027) (Fig. 2), this difference failed to reach significant [*t*(58) = 0.270, *p* = 0.788].

**Figure 3:**
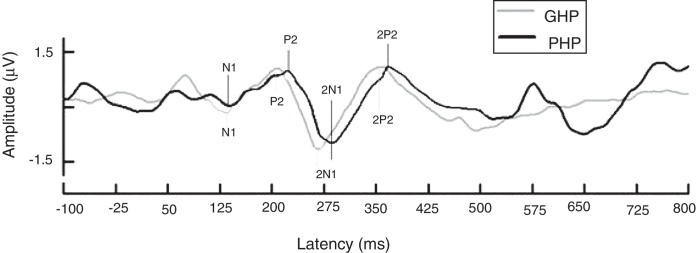
Grand average waveform of ACC obtained from GHP and PHP. The latency of ACC is earlier in GHP than PHP. This is true for both sub-groups.

Further, in order to know if there was any significant difference between groups (with two factors as good and poor hearing aid performers) on each latency (4 levels) and amplitude (4 levels) component of ACC, a MANOVA was performed and there were 4 levels each in latency and amplitude of ACC. From Fig. 3, it was observed that except N1 latency and 2P2 amplitude, the latency of each component of ACC was significantly earlier and amplitude was higher in good hearing aid performers than poor hearing aid performers (Table 1).

**Table 1:** Mean, standard deviation (SD) and MANOVA results with df (1, 58) on latency and amplitude of ACC.

Components of ACC		Latency (ms)	*F* value	*p*-Value	Amplitude (μV)	*F* value	*p*-Value
	Groups	Mean ± SD			Mean ± SD		
N1	GHP	148.08 ± 16.08	0.18	0.671	−0.68 ± 0.52	8.39	0.005
	PHP	146.52 ± 10.07			−1.05 ± 0.44		

P2	GHP	228.94 ± 7.92	13.78	0.000	1.17 ± 0.54	5.19	0.026
	PHP	242.84 ± 7.07			0.82 ± 0.65		

2N1	GHP	285.97 ± 7.92	10.61	0.002	−1.58 ± 0.62	8.10	0.006
	PHP	292.92 ± 8.51			−1.34 ± 0.59		

2P2	GHP	375.60 ± 13.32	19.84	0.000	1.46 ± 0.74	2.39	0.127
	PHP	390.36 ± 11.64			0.93 ± 0.63		

**Figure 4:**
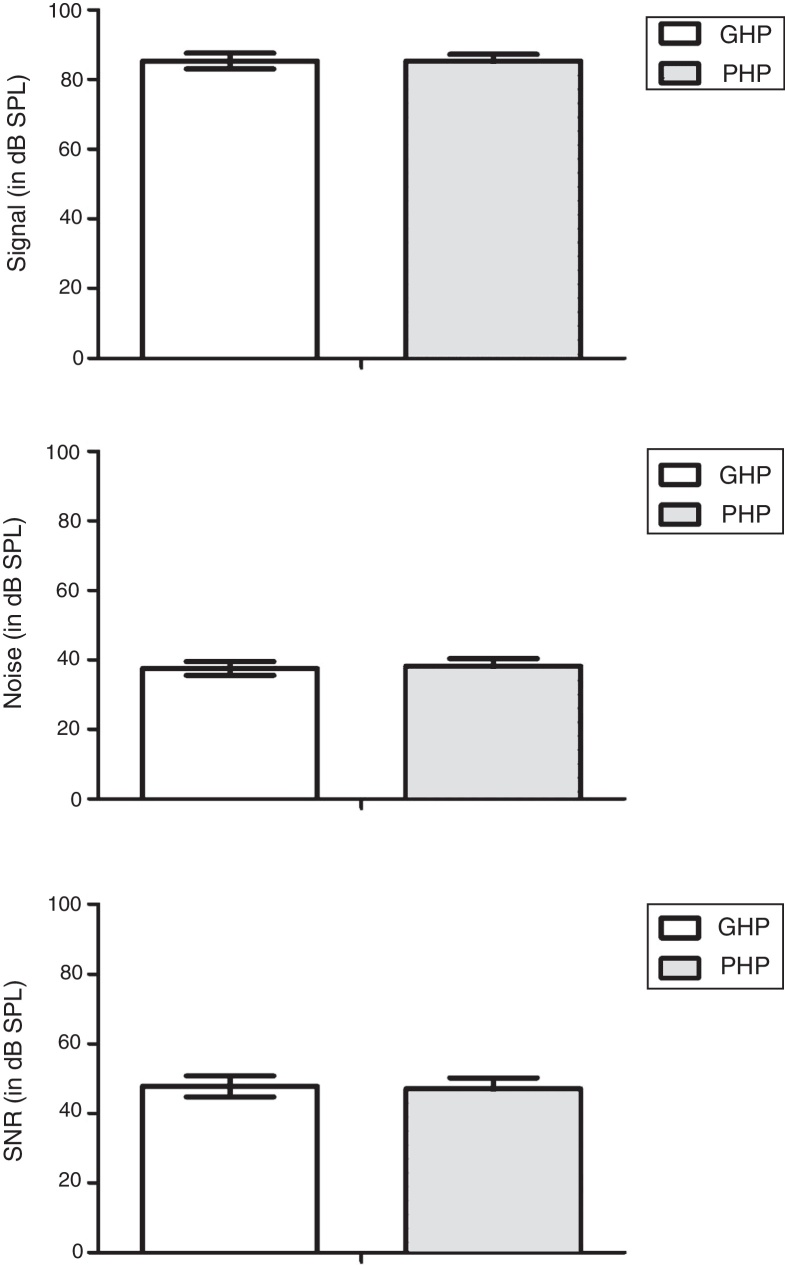
Bar graph showing signal level, noise level and signal to noise ratio of amplified speech /si/ from GHP and PHP groups.

It is well established that acoustic change complex is an exogenous potential and it is influenced by signal characteristics. Thus, signal level, noise level and signal to noise ratio were compared between groups to probe whether signal characteristics have influenced on the representation of amplified speech at auditory cortical level. The data of signal level, noise level and signal to noise ratio of amplified speech were obtained from GHP and PHP was subjected to a separate independent sample t test. The results revealed that there was no significant difference between groups in the signal level [*t*(58) = 0.10, *p* = 0.450]; noise level [*t*(58) = −1.33, *p* = 0.771]; and signal to noise ratio [*t*(58) = 0.97, *p* = 0.633]. These results suggest that signal characteristics of amplified speech are same in both groups (Fig. 4).

**Figure 5:**
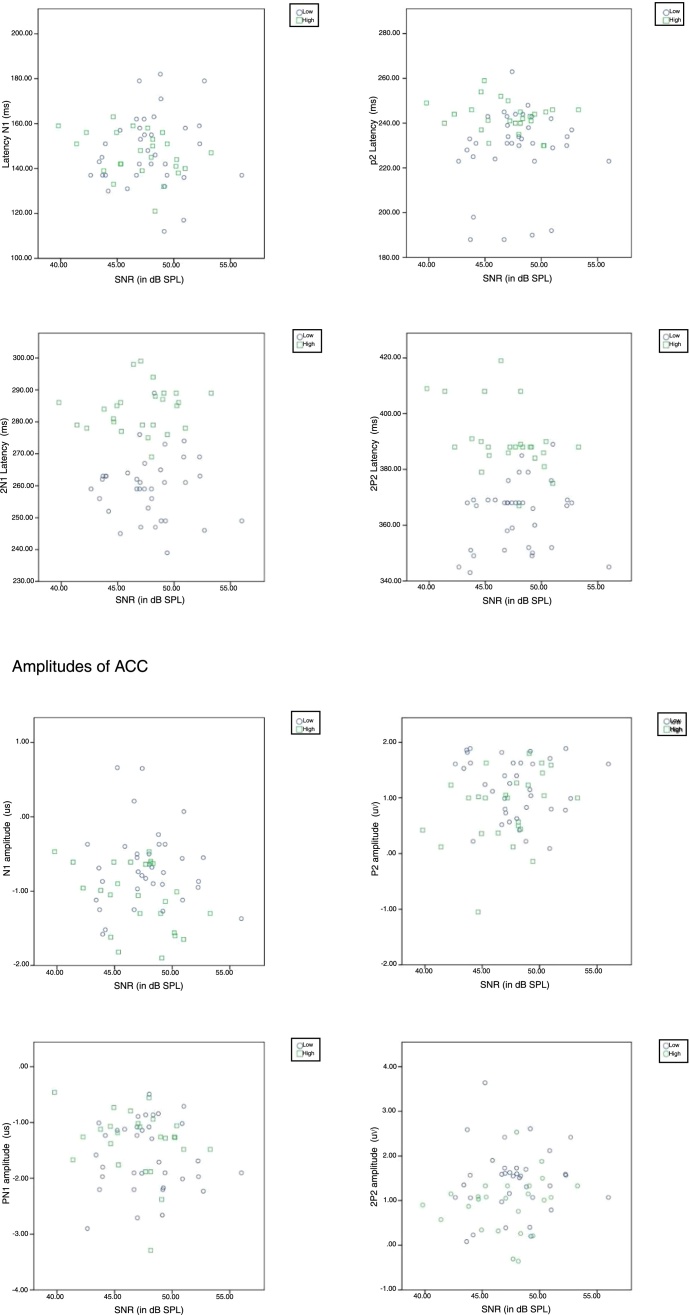
Correlation between signal to noise ratio and latency; amplitude of ACC.

Further to determine the influence of SNR on any of the component of ACC, a Pearson product moment correlation was performed. The result revealed that, there was no significant relationship (*p* > 0.05) between the SNR and each component of the latency, the amplitude of ACC (Fig. 5). It indicates that a change in a SNR has not caused a significant effect on each component of ACC.

To ascertain difference in representation of amplified speech at auditory cortical level from GHP and PHP has any influences on speech perception, the aided SNR 50 was evaluated from them. The aided SNR 50 in dB was obtained from participants of each group. It was subjected to an independent samples *t* test to evaluate difference between GHP (Mean = 3.21, SD = 2.65) and PHP (Mean = 3.50, SD = 2.54) on aided SNR 50. The test result revealed that there was no statistically significant [*t*(58) = 1.32, *p* = 0.212] between groups on SNR 50.

**Figure 6:**
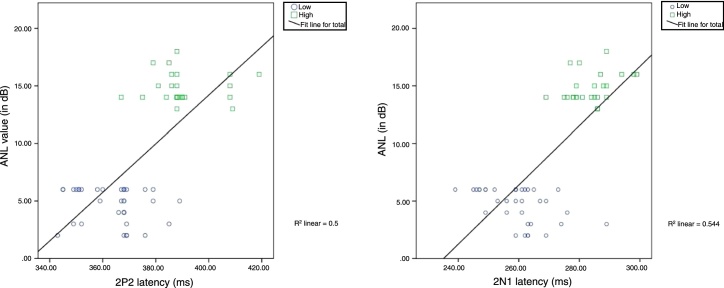
Linear regression drawn with measured data and mean of the predicted data for 2N1 and 2P2 on a scatter plot. The predicted data a ANL score increases linearly with the increase in the latency of either 2N1 or 2P2.

Further, to assess the correlation between raw ANL scores of the study participants (dependent variable) and the latency and amplitude of ACC, EDI for each stimulus, SNR 50, and GDT, a series of Pearson product moment correlation were carried out. The results showed a significant strong positive correlation for latency of 2N1 (*r* (60) = 0.738, *p* = 0.000) and 2P2 (*r* (60) = 0.704, *p* = 0.000) with increase in raw ANL scores. Before performing multiple regressions, multicollinearity was assessed between independent variables i.e., latencies of 2N1 and 2P2. These independent variables are significantly correlated and its strength found to be strong with the values of *r* = 0.700 and *p* = 0.000. Thus, a linear regression was performed to predict acceptable noise level from latencies of 2N1 and 2P2. The regression line was fitted in the scatter plot as shown in Fig. 6 and equations *y* = *β* (*x*) − *β*_0_ [*β*_0_ = 60.52, *β* = 0.25, *r*^2^ = 0.544] and *y* = *β* (*x*) − *β*_0_ [*β*_0_ = −70.28, *β* = 0.21, *r*^2^ = 0.50] were obtained for the latencies of 2N1 and 2P2 respectively to predict the raw ANL score. The results indicate that with an increase in the latency of 2N1 or 2P2, the raw ANL score increases linearly.

**Figure 7:**
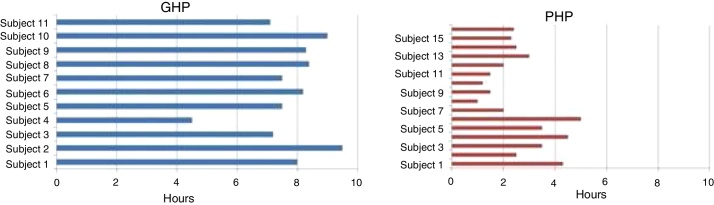
Time line graph showing average hearing aid usage in a day from the follow up participants of GHP and PHP groups.

Good hearing aid performers and poor hearing aid performers constitute 58% (*n* = 35) and 48% (*n* = 25) respectively. Using data logging option, the hearing aid usage was assessed on eleven participants of GHP (32%) and 16 participants of PHP (64%) who were visited the clinic for a follow up. Data logging analysis revealed that GHP participants have used their hearing aid on an average of 7.2 h per day. Whereas, PHP participants used their hearing aid less than 3 h in a day (Fig. 7).

## Discussion

Temporal resolution was impaired in both groups reflected in GDT is in consonance with the study by Hoover et al.^21^ who reported GDT of 9 ms in their study participants of older adults with hearing impaired. The hearing aid fitted to the participants of GHP and PHP showed minimal temporal alteration. This could be due to the decreased modulation depth in the aided condition due to compression in different channels of the hearing aid. Further, signal level, noise level and signal to noise ratio were analyzed and it was found that values analyzed were almost same between groups. This could be due to the gain provided by the aid was almost same as the degree of hearing loss was almost same across study participants. Moreover, stimulus /si/ was presented at 65 dB SPL. In addition, equivalent input noise (EIN) of 9–10 dB was noticed as same company hearing aid was recommended to procure. To ascertain how available cues in speech stimuli after hearing aid processing are represented at cortical level from both groups, LLR and ACC were recorded and compared between good and poor hearing aid performers. The cortical responses were absent in the unaided condition as the stimuli were not audible. Whereas in aided condition, it was observed that except slope of N1-P2 complex of LLR, N1 latency and 2P2 amplitude of ACC, the latency of each component of ACC was significantly earlier and amplitude was higher in good hearing aid performers than poor hearing aid performers. It infers for detection of sound a neural representation was similar between groups but for identifying the subtle cues in an ongoing speech requires strong excitatory and inhibitory neural response.^22^ In addition, the difference exists between group on ACC could be due to greater depolarization^23^ that leads to faster neural conduction^24^ at the cortical level. In addition, stronger efferent feedback might have played an instrumental role for earlier processing and increased strength^25^, ^26^, ^27^, ^28^, ^29^ in GHP. It suggests the central afferent and efferent mechanisms are more active in GHP.

Now the question arises is how sure is that the differences in ACC between groups are not influenced by stimulus characteristics? It is well established fact that ACC are exogenous potentials which is influenced by stimulus characteristics. Whether the signal to noise ratio of amplified speech caused the difference among the groups? Thus, we have correlated the SNR with each latency and amplitude of ACC. It was found that signal to noise ratio was found to be almost same and its influence on each component of ACC was negligible. Further, correlation was conducted between ANL and latency; amplitude of ACC. The latencies of 2N1 and 2P2 were strongly related with ANL values. Further, a regression analysis was performed to predict ANL score from the latencies of 2N1 and 2P2. It infers a 1 ms change in latencies of either 2N1 or 2P2 causes the raw ANL score to increase by 0.25 dB and 0.21 dB respectively, with the prediction percentage of 54%. This indicate that ANL is physiologically sensitive in keeping physical characteristics of stimulus were found to be same across groups.

Yet another question is whether this subtle difference at cortical response between groups has influenced on speech perception in noise? A SNR 50 was evaluated from participants of study. It was observed that mean SNR 50 in GHP was 3.21 dB and in PHP was 3.50 dB but this difference did not reach significant difference. It infers that an almost 3–3.5 dB higher signal level was required than noise level to obtain 50% recognition level in both groups. To be clear each group of participants have merely repeated the sentences in the presence of noise to obtain 50% at 3–3.5 dB SNR. Though both groups had similar SNR 50 the annoyance level toward noise in them has reflected at auditory cortical level. It indicates that though the study participants have obtained similar aided benefit as reflected from the score of SNR 50, hearing aid usage is depend on annoyance level toward background noise. That is the hearing aid usage is influenced by accepting the annoyance toward the ambient noise amplified by aid or being able to put up the hearing aid circuitry noise other than speech perception in noise.

The hearing aid usage from data logging was obtained from the study participants who were instructed to come to the hearing clinic for a follow up. About 64% of PHP (16) and 32% (11) of GHP participants visited the clinic. The participants of PHP have reported unsatisfied with the hearing aid because of background noise. On data logging analysis revealed that PHP participants have used their hearing aid less than 3 h in a day. Whereas GHP participants reported a satisfactory benefit from their aid and data logging analysis found that they have used their hearing aid on an average of 7.2 h per day. It infers that annoyance toward noise have restricted to use their hearing aid for a long hour in PHP participants. Thus, ANL measurement should be performed at least before procuring a hearing aid or at the time of fitting the hearing aid such that appropriate well fitted hearing aid with suitable options can be selected.

The findings of the study accept alternate hypothesis. Though study participants have similar temporal ability with minimally altered temporal contrasts in speech stimuli after processed through hearing aid, the annoyance level reflected subtle difference in the representation of amplified speech at cortical level from good and poor hearing aid performers. To conclude for those individuals who have a high ANL score tend to have likelihood chance of either reject their own hearing aid or become a part time hearing aid users even though their speech perception in noise is good.

## Conclusion

Though the hearing loss and temporal resolution are same between good and poor hearing aid performers a subtle difference on aided representation of amplified speech at auditory cortical level is mediated by annoyance toward noise, which decides usage of hearing aid.

## Ethical approval

All procedures performed in studies involving human participants were in accordance with the ethical standards of the AIISH institutional research committee.

## Informed consent

Informed consent was obtained from all individual participants included in the study.

## Conflicts of interest

The authors declare no conflicts of interest.
